# Commentary: Modulation of Prepulse Inhibition and Startle Reflex by Emotions: A Comparison between Young and Older Adults

**DOI:** 10.3389/fnagi.2016.00106

**Published:** 2016-05-09

**Authors:** Mara Mather

**Affiliations:** Leonard Davis School of Gerontology, University of Southern California, Los AngelesLos Angeles, CA, USA

**Keywords:** startle reflex, prepulse inhibition, emotion, negative valence, aging, eyeblink response, pictures, positivity effect

Some findings overturn basic assumptions in the field and make it obvious how little we really understand. In my opinion, findings by Le Duc et al. (2016) regarding age differences in how emotional stimuli influence startle reactivity fit this profile. They found age differences in how viewing pleasant, unpleasant and neutral pictures modulated a startle response to a loud noise (Figure 1A). While the younger adults (ages 20–29) startled most to loud noises while viewing unpleasant pictures, the older adults (ages 56–69) startled least while viewing the unpleasant pictures.

**Figure 1:**
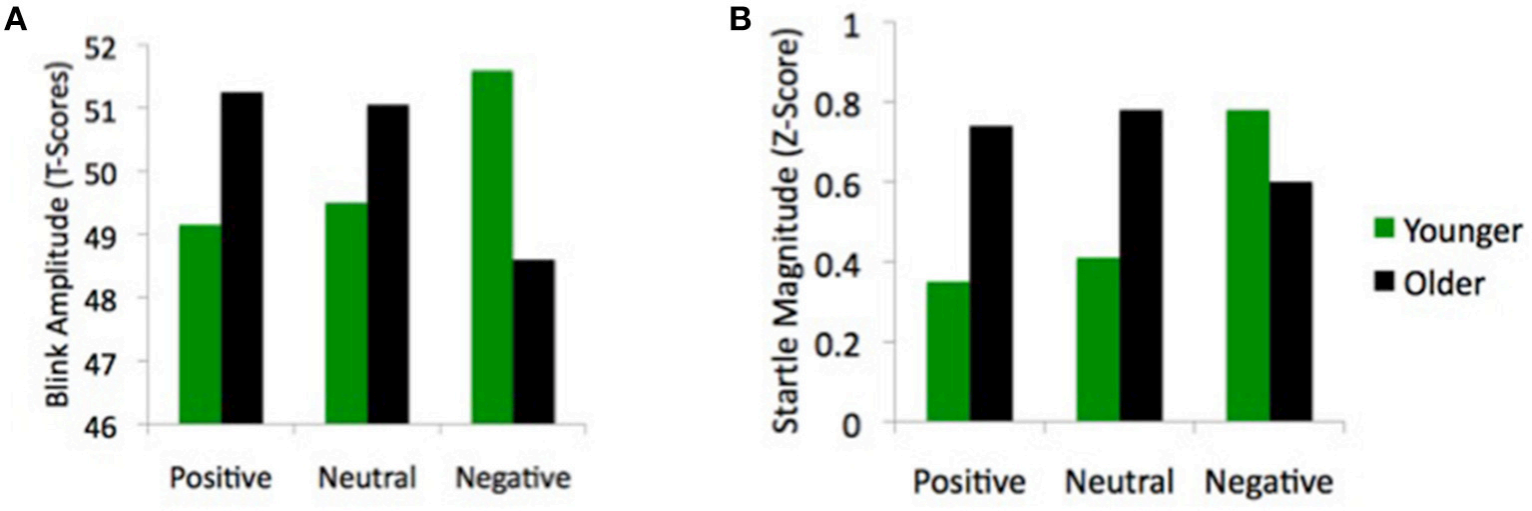
**Age-by-valence interactions in startle reactivity from (A) Le Duc et al. (2016); and (B) Feng et al. (2011)**.

The pattern Le Duc et al. found with younger adults is consistent with many previous studies in which undergraduates startled more to loud noises while viewing unpleasant pictures than while viewing pleasant pictures, with neutral pictures yielding startle levels in between the two valences (Vrana et al., 1988; Bradley et al., 1990, 1996; Cook et al., 1992; Stritzke et al., 1995; Cuthbert et al., 1996; Stanley and Knight, 2004; Larson et al., 2005; Gard et al., 2007; Ruiz-Padial et al., 2011). Five-month-old infants showed the same pattern when viewing emotional faces (Balaban, 1995). Thus, this pattern of greater startle during exposure to negative than positive stimuli is a highly replicated finding. Lang (1995) proposed that startle is a defensive reaction augmented during aversive motivational states and dampened during approach states. This is now the dominant assumption in the field (e.g., Crowell et al., 2015).

However, Le Duc et al.'s findings with older adults contradict this dominant assumption and reveal that we lack basic understanding about how emotion influences startle. Importantly, their surprising finding is not just a one-off finding. It replicates findings from another study comparing startle reactivity in younger adults (age 18–23) vs. older adults (ages 65–88) (Feng et al., 2011; Figure 1B). Thus, across two studies, older adults' results contradict the Lang (1995) model. Other than these two studies, I am not aware of any other emotion-startle data with older adults.

From the perspective of the aging literature, it is not surprising that older adults would show a different impact of emotional pictures than younger adults. Previous findings indicated that younger and older adults show opposing affective biases in attention and memory, with younger favoring negative and older favoring positive relatively more (Mather and Carstensen, 2005; Reed et al., 2014). If Le Duc's finding had been a matter of degree (e.g., older adults showed less of an increase in startle during negative pictures than did younger adults), we could have accounted for the age differences within Lang's (1995) rubric. However, Le Duc et al.'s findings are more interesting than a simple age-related diminishment in the effect: the affective-modulated startle effect actually reversed itself among the older adults. Thus, the older adults' results contradict the notion that startle is a defensive reaction augmented during aversive motivational states and dampened during approach states (Lang, 1995).

In addition to replicating Feng et al.'s findings of an age-related reversal of the effects of negative pictures on startle, Le Duc et al. also found age differences in how affective pictures modulated prepulse inhibition effects. Prepulse inhibition occurs when a non-startling mild stimulus (the prepulse) attenuates the startle reflex to a subsequent intense startling stimulus (Li et al., 2009). The linear pattern of effects was similar across age groups (positive > neutral > negative), but where the effects were largest differed across age groups. Younger adults showed significantly reduced prepulse inhibition for negative pictures than neutral or positive pictures, whereas older adults showed the greatest difference for positive pictures, with more prepulse inhibition for positive than neutral or negative pictures.

These findings are not only interesting in regards to age differences in emotion processing but they also raise fundamental questions about the mechanisms of startle. What processes could account for both younger and older adults' pattern of findings? There are a number of possibilities that merit future investigation. One possibility favored by Feng et al. in their discussion is that the degree of attentional focus on the affective picture could influence startle. However, given this hypothesis, it is surprising that there was not also an age reversal in patterns for the impact of valence on prepulse inhibition. Another possibility, as raised by Le Duc and colleagues, is that the amygdala has a key role in potentiating or suppressing startle, and because younger and older adults have opposite amygdala responses to negative vs. positive pictures (Mather et al., 2004; for review see Mather, 2016), valence also has opposing effects on startle. Recent research also indicates that prefrontal cortex contributes to younger adults' diminished startle during viewing positive pictures (Hurlemann et al., 2015); thus age differences in prefrontal responses to emotional stimuli (Mather, 2012; Nashiro et al., 2012) may modulate startle reactivity. Also potentially relevant is that engaging emotion down-regulation can decrease reactivity whereas up-regulation increases it (e.g., Conzelmann et al., 2015). Left to their own devices, older adults may engage down-regulate responses to negative and upregulate responses to positive pictures whereas younger adults may do the opposite. Another interesting avenue worth exploring are findings suggesting that increased phasic dopamine transmission increases startle potentiation by negative pictures (Domschke et al., 2015), as older adults show declines in dopaminergic function (Bäckman et al., 2010).

In summary, the opposing findings from younger vs. older adults indicate that the effects of emotion on startle cannot be predicted from valence alone and that there is some other mechanism driving the effect that leads to reversed outcomes in later life. There are a number of possibilities worth exploring and better understanding of these age differences will hopefully shed light on the basic mechanisms of startle reactivity. More generally, these findings illustrate a case in which studying aging contributes to our understanding not just of developmental change, but also the basic mechanisms of a process within the young adult population.

## Author contributions

The author confirms being the sole contributor of this work and approved it for publication.

## Funding

Preparation of this paper was supported by NIH grant RO1AG025340.

### Conflict of interest statement

The author declares that the research was conducted in the absence of any commercial or financial relationships that could be construed as a potential conflict of interest.
